# Update on COVID-19 Myocarditis

**DOI:** 10.3390/medicina56120678

**Published:** 2020-12-09

**Authors:** Arianne Clare C. Agdamag, Jonathan B. Edmiston, Victoria Charpentier, Mohammed Chowdhury, Meg Fraser, Valmiki R. Maharaj, Gary S. Francis, Tamas Alexy

**Affiliations:** 1 Division of Cardiology, Department of Medicine, University of Minnesota, Minneapolis, MN 55455, USA; agdam001@umn.edu (A.C.C.A.); mfraser10@umphysicians.umn.edu (M.F.); maha0104@umn.edu (V.R.M.); franc354@umn.edu (G.S.F.); 2 Department of Medicine, University of Minnesota, Minneapolis, MN 55455, USA; blair.edmiston@gmail.com (J.B.E.); charp024@umn.edu (V.C.); 3 Cardiology, North Central Heart, Sioux Falls, SD 57108, USA; MChowdhury@ncheart.com

**Keywords:** COVID-19, myocarditis, coronavirus, heart failure, cardiogenic shock

## Abstract

Severe Acute Respiratory Syndrome Coronavirus 2 (SARS-CoV-2) gained worldwide attention at the end of 2019 when it was identified to cause severe respiratory distress syndrome. While it primarily affects the respiratory system, we now have evidence that it affects multiple organ systems in the human body. Cardiac manifestations may include myocarditis, life threatening arrhythmias, acute coronary syndrome, systolic heart failure, and cardiogenic shock. Myocarditis is increasingly recognized as a complication of Coronavirus-19 (COVID-19) and may result from direct viral injury or from exaggerated host immune response. The diagnosis is established similar to other etiologies, and is based on detailed history, clinical exam, laboratory findings and non-invasive imaging studies. When available, cardiac MRI is the preferred imaging modality. Endomyocardial biopsy may be performed if the diagnosis remains uncertain. Current management is mainly supportive with the potential addition of interventions recommended for severe COVID-19 disease, such as remdesivir, steroids, and convalescent plasma. In the setting of cardiogenic shock and refractory, life-threatening arrhythmias that persist despite medical therapy, advanced mechanical circulatory support devices should be considered. Ultimately, early recognition and aggressive intervention are key factors in reducing morbidity and mortality. Our management strategy is expected to evolve further as we learn more about COVID-19 disease and the associated cardiac complications.

## 1. Introduction

Coronavirus-19 (COVID-19) disease is caused by the Severe Acute Respiratory Syndrome Coronavirus 2 (SARS-CoV-2). This novel virus gained worldwide attention in December 2019 after it was isolated and identified to cause severe acute respiratory distress syndrome in a cluster of patients in China’s Hubei Province [1]. The World Health Organization declared COVID-19 a global pandemic on 11 March 2020 [2]. While initially thought to primarily target the respiratory system, it soon became evident that infection with SARS-CoV-2 affects multiple organ systems in the human body. The cardiovascular system is no exception. Recognizing cardiac involvement is critically important as it portends a significantly elevated risk of in-hospital mortality (51% vs. 4%) and adversely impacts long-term clinical outcomes [3]. Myocarditis is now a recognized severe complication of COVID-19 prompting numerous research groups to focus on developing early diagnostic approaches and management strategies. The primary goal of this article is to provide a review of COVID-19 disease with special focus on myocarditis.

## 2. Overview of SARS-CoV-2

SARS-CoV-2 belongs to the β subgroup of coronaviruses, along with SARS-CoV that was initially reported in China’s Guangdong Province in 2003, and the Middle East Respiratory Syndrome Coronavirus, MERS-CoV, first described in 2012 [4,5,6]. Genomic sequencing revealed a 93.1% identity between SARS-CoV-2 and the coronavirus RaTG12 isolated from the common horseshoe bat (*Rhinolophus affinis*) in Yunnan, China, in 2013. This suggests that this zoonotic pathogen endemic to bats, perhaps, crossed the species barrier to infect humans, similar to other viruses in the past [7,8]. Following its initial transfer, SARS-CoV-2 achieved a human-to-human transmission rate significantly higher than that documented with other β coronaviruses [4]. It is a positive, single-stranded RNA virus with a glycoprotein envelope and a large number of polysaccharide-coated spike [S] proteins covering its surface. Located within the S1 subunit is the receptor binding domain that is responsible for attaching to the host cell angiotensin converting enzyme 2 (ACE2) receptors thereby facilitating viral entry into the target cells [9]. ACE2 is expressed abundantly on type II pneumocytes as well as in cardiovascular, renal, and gastrointestinal tissues which accounts for the pathogenesis, multi-organ involvement and clinical manifestations of COVID-19 disease [10]. While multiple possible human-to-human transmission routes have been identified, spreading via aerosol and droplets are thought to be the most common [11]. Infected individuals may develop an array of mild to moderate symptoms, such as fever, dry cough, shortness of breath, fatigue, weakness, headache, sore throat, and loss of taste or smell [10,12,13]. Patients with underlying comorbidities including hypertension, diabetes mellitus, obesity, cardiovascular disease, pulmonary disease, and cancer have a higher likelihood progressing to more severe illness culminating in multi-organ failure [4]. Notably, 40% to 45% of individuals may remain completely asymptomatic and unaware of their infection and can transmit the virus for an extended period of time [14]. Common laboratory findings in COVID-19 disease include lymphopenia, thrombocytopenia, elevated serum troponin, severely elevated inflammatory markers, high d-dimer, and abnormal renal and liver function tests [10,15,16]. Pulmonary involvement is usually evident on routine chest X-ray and may present as bilateral ground glass opacities in the posterior and peripheral lung fields on CT scan [4,10,17,18]. These imaging findings often correspond to diffuse alveolar hemorrhage with interstitial inflammation, hyaline membrane formation, and widespread microangiopathy with thrombosis [10,19]. Recognizing cardiac involvement may be more challenging but is of utmost importance given its adverse impact on clinical outcomes [3]. The injury may result from various mechanisms, including direct myocardial damage caused by SARS-CoV-2, electrolyte abnormalities, relative ischemia, coronary thrombosis, acute plaque rupture, or cytokine storm [20]. Consequently, cardiac manifestations may be broad and include signs and symptoms of acute heart failure, cardiogenic shock, myocarditis, acute coronary syndrome, a broad range of atrial and ventricular arrhythmias, and cardiac arrest [3,21]. The following sections will focus primarily on myocarditis, a potentially severe and lethal complication of COVID-19 infection.

## 3. Pathophysiology of COVID-19 Associated Myocarditis

Myocarditis refers to the inflammation of the cardiac muscle due to a variety of infectious and non-infectious diseases [22]. Viral etiology remains the leading cause of myocarditis in the United States and has been documented as a complication in patients infected with enterovirus including coxsackievirus, parvovirus B-19, H1N1 and members of the β coronavirus group, including MERS [23,24,25,26]. The exact pathophysiology of SARS-CoV-2-associated myocarditis remains elusive at this time. Depending on host-related factors (i.e., virus-host interactions) and the phase of infection (acute, subacute, or chronic), proposed mechanisms may include: (1) immune-mediated, (2) autoimmune-mediated, and (3) direct virus-induced [27]. In immune-mediated myocarditis, both innate and acquired immune responses contribute to myocardial injury with the sequelae of dilated cardiomyopathy [28,29]. Despite this mechanism, large trials with immunosuppressive therapies such as prednisone and azathioprine failed to show significant clinical benefit; however, there is some evidence that other immunomodulation strategies may be effective [30,31,32,33,34]. Autoimmune-mediated myocarditis may develop in response to the release of cryptic antigens from cardiac myocytes that are normally secluded from the immune system following virus-mediated injury [35]. There is also evidence to support the hypothesis that molecular mimicry involving epitopes shared among viral capsid proteins, cardiac myosin, and other unidentified proteins on the surface of cardiac myocytes stimulate autoimmune reactions [36]. When viruses evade the innate immune system, they replicate and create viral proteins that cause direct myocardial injury by promoting cellular apoptosis and necrosis [37]. SARS-CoV-2 likely causes myocarditis in humans through one of the pathways similar to other viral pathogens [26,38]. While MERS-CoV utilizes a protein called 4a-accessory protein to impair cellular stress granule formation, SARS-CoV enhances its RNA translation via the Nsp1 protein [39,40]. As discussed previously, SARS-CoV-2 may invade cardiac myocytes using their surface ACE2 receptors and may cause direct cellular damage. The presence of viral genome was not definitively associated with increased mononuclear infiltration within the myocardium [41].

## 4. Clinical Presentation of Patients with COVID-19 Myocarditis

Similar to other viral infections, the most common initial presentation of COVID-19 includes fatigue, fever, chills, cough, and gastrointestinal complaints. Symptoms may remain mild even if myocarditis ensues; however, a subset of patients develop severe disease manifestations including chest pain, life-threatening arrhythmias, and cardiogenic shock [24]. At this time, it is not possible to accurately predict which patients will develop severe cardiac complications, and the exact incidence of COVID-19-associated myocarditis remains unknown [42]. Historical epidemiological data suggest that young individuals and males are more susceptible to develop myocarditis. It remains to be seen if these hold true for COVID-19-associated cases [23,43].

## 5. Establishing the Diagnosis of Myocarditis

Unfortunately, no single laboratory test exists to establish the diagnosis of myocarditis. While increased serum troponin in COVID-19 infection may identify patients with severe disease, myocardial involvement, and increased risk of mortality, elevated levels are not specific for myocarditis and may reflect supply/demand mismatch [44]. Natriuretic peptides and inflammatory markers such as ESR and CRP are neither sensitive nor specific [23]. Elevated liver enzymes, serum creatinine, and lactic acid may suggest end organ dysfunction and hypoperfusion related to cardiogenic shock although none of these are specific.

Routine electrocardiogram (ECG) is typically abnormal in patients with myocarditis. Common findings include rhythm disturbances, low QRS voltage due to myocardial edema, ST-segment and T-wave changes, as well as conduction abnormalities [24,45]. However, none of these abnormalities are sensitive or specific enough to establish the diagnosis of myocarditis and are thought to be related to direct myocardial injury caused by SARS-CoV-2, hypoxia, and COVID-19 associated systemic inflammatory response [46,47].

Transthoracic echocardiography (TTE) is the initial non-invasive imaging modality of choice to evaluate for COVID-19-associated cardiac complications. Findings may include global left ventricular (LV) or biventricular dysfunction, myocardial edema, LV thrombus, and pericardial effusion [24]. TTE may also reveal other cardiac abnormalities, such as regional wall motion abnormalities suggesting myocardial ischemia or valvular disease. While ultrasound operators are certainly at increased risk for disease transmission given the need for close patient contact, in our experience, the study may be performed serially and safely with appropriate personal protective equipment to monitor for cardiac recovery.

Cardiac magnetic resonance imaging (CMR) is an excellent imaging modality to assess biventricular structure and function. When interpreted by an experienced clinician, CMR provides tissue-level diagnosis including myocardial edema and interstitial fibrosis [24]. The Lake Louise Criteria (LLC) was revised in 2018 and requires both of these criteria to diagnose myocarditis: (1) myocardial edema on T2-mapping of T2W images and (2) non-ischemic myocardial injury signified by abnormal T1, extracellular volume, or late gadolinium enhancement [48]. Findings in COVID-19 related acute myocarditis do not differ from those described in the LLC [49]. Due to the exam duration and logistics, CMR should primarily be considered for hemodynamically stable patients.

**Figure 1:**
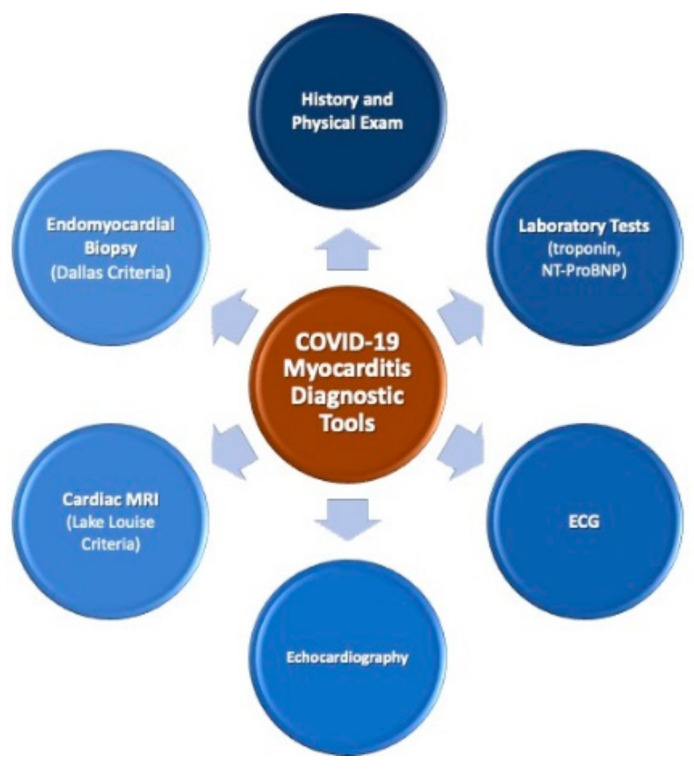
Tools used to diagnose COVID-19 myocarditis. COVID-19: Coronavirus-19; ECG: Routine 12-lead electrocardiogram.

Endomyocardial biopsy (EMB) is often considered to aid in the diagnosis of myocarditis. The 1987 Dallas criteria require lymphocytic infiltration associated with myocyte injury in the absence of ischemia [50]. While highly specific, reported sensitivity for myocarditis diagnosed with EMB is between 10% and 22%. The low sensitivity is attributed to sampling error caused by the often patchy myocardial involvement and high interobserver variability [51,52]. A large panel of monoclonal antibodies (including anti-CD3, T lymphocytes, anti-CD68, macrophages, and anti HLA-DR) is needed to identify and characterize the inflammatory infiltrate [23]. EMB may be enhanced by DNA-RNA extraction and RT-PCR to amplify and detect the presence of the viral genome [53]. Initial case reports failed to reliably demonstrate the direct invasion of cardiomyocytes by SARS-CoV-2 and current ESC guidelines do not recommend cardiac biopsy for COVID-19 patients with suspected myocarditis [54]. More recent studies, however, were able to detect the viral genome in some patients from EMB samples as well as during post mortem evaluation of cardiac tissue [41,55,56]. The presence of cellular infiltrates and necrosis was highly variable and autopsy findings did not demonstrate a specific, reproducible histopathology of COVID-19 myocardial insult [57]. Further studies are needed to define which patients may benefit from invasive evaluation using EMB and to establish the ideal timing for the biopsy. Figure 1 summarizes the currently utilized tools in the diagnosis of COVID-19 myocarditis.

## 6. Patient Management

Due to the novelty of the disease and the lack of randomized clinical trial data, the management of myocarditis secondary to COVID-19 has largely been similar to other myocarditis etiologies, that is, providing supportive care. While no specific treatment strategies have been found efficacious, many are currently under investigation [54]. Table 1 summarizes current management of COVID-19-associated myocarditis.

The recently approved remdesivir has gained traction early in the United States owing to a trial showing faster recovery times in hospitalized patients, prompting the Food and Drug Administration to grant Emergency Use Authorization for severe infection on 1 May 2020 [58]. Studies with convalescent plasma, interferon beta, dexamethasone, and hydrocortisone are ongoing [59,60,61]. Results with dexamethasone have shown promise for the treatment of severe COVID-19 infection, as published in the RECOVERY trial [62]. Although steroid administration for viral myocarditis remains highly controversial, it is frequently employed and may help blunt the immune response in case of COVID-19. Disease severity, comorbidities and reviewing possible drug toxicities must drive the individualized decision to administer these medications.

Patients that develop heart failure from COVID-19 myocarditis should be treated with guideline-directed medical therapy, including angiotensin converting enzyme inhibitors (ACEi), angiotensin receptor blockers (ARB), or angiotensin receptor-neprilysin inhibitors (ARNi), beta blockers, mineralocorticoid receptor antagonists, and diuretics as clinically indicated [54]. Owing to their mechanism of action, there was initial concern that medications in the ACEi, ARB and ARNi group could worsen clinical outcomes in patients infected with COVID-19. However, a recent observational study did not demonstrate any harm [63,64]. As such, it is generally recommended to initiate or continue these medications during the acute phase of the disease, and beyond as indicated and tolerated [65,66,67].

Arrhythmias are common in myocarditis, independent of the underlying etiology. Beta blockers approved for the management of heart failure may be considered for hemodynamically stable patients. Amiodarone is typically the antiarrhythmic agent of choice in the critically ill, although it can prompt QTc prolongation, especially when combined with azithromycin or hydroxychloroquine [68]. Alternatively, lidocaine infusion or oral mexiletine may be considered.

The International Society of Heart and Lung Transplantation (ISHLT) COVID-19 task force has recently released the recommendation to consider advanced therapies for affected patients if indicated [69]. Patients with fulminant myocarditis may continue to decompensate and develop cardiogenic shock or malignant arrhythmias. Ultimately, they may require the use of inotropes, intra-aortic balloon pump, or other temporary mechanical circulatory support devices to stabilize their hemodynamics. Regarding extracorporeal membrane oxygenation (ECMO), providers should rely on local expertise while optimizing resource utilization [70]. The Extracorporeal Life Support Organization (ELSO) has also released a guidance document on when to consider ECMO support in this population [71]. The active registry managed and maintained by the organization monitors outcomes as well as complications.

## 7. Prognosis of COVID-19 Myocarditis

There are very limited data currently on the prognosis of COVID-19 myocarditis. Patients with pre-existing cardiac conditions and those with elevated serum troponin during their disease course have worse outcomes, including higher mortality, longer length of hospital stay and need for mechanical ventilation [44,72]. Patients who develop heart failure will require guideline-directed medical therapy and long-term follow-up with a multidisciplinary team. The minimum duration of medical therapy remains to be determined.

## 8. Conclusions

Myocarditis is one of the serious cardiac complications of the SARS-CoV-2 infection. Owing to its highly variable clinical presentation, the exact incidence of the disease remains unknown at this time. It has a potentially lethal clinical course and several case reports have highlighted the importance of aggressive screening measures in high-risk populations to establish the diagnosis in a timely manner. Although cardiac MRI is the non-invasive modality of choice to diagnose myocarditis, history, physical examination, laboratory evaluation and the widely available cardiac echocardiography are indispensable in raising suspicion and prompting further evaluation. While no specific guidelines are available on the management of myocarditis secondary to COVID-19, aggressive supportive measures, including the use of temporary mechanical circulatory support devices, may be necessary. Hemodynamically stable patients should be initiated on guideline directed medical therapy for heart failure. Further research is needed to better understand long-term risks and complications.

## Figures and Tables

**Table 1:** Management of COVID-19-associated myocarditis. ACEi (Angiotensin Converting Enzyme inhibitor), ARB (Angiotensin II Receptor Blocker), ARNi (Angiotensin Receptor-Neprilysin Inhibitor), VV-ECMO (Veno-Venous Extracorporeal Membrane Oxygenation), VA-ECMO (Veno-Arterial Extracorporeal Membrane Oxygenation).

MANAGEMENT OF COVID-19 ASSOCIATED MYOCARDITIS
**Supportive Cares** **COVID-19 specific treatment:** Remdesivir
**Investigational treatment options:** Convalescent plasma, interferon beta, steroids
**Guideline-directed medical therapy for heart failure:** Beta blockers, ACEi/ARB/ARNi, mineralocorticoid receptor antagonists, diuretics
**Patients with hemodynamic compromise:** Inotropes, temporary mechanical circulatory support devices, VV-ECMO, VA-ECMO
**Close follow-up with multidisciplinary team**

## References

[1] World Health Organization Naming the Coronavirus Disease (COVID-19) and the Virus that Causes It. https://www.who.int/emergencies/diseases/novel-coronavirus-2019/technical-guidance/naming-the-coronavirus-disease-(covid-2019)-and-the-virus-that-causes-it.

[2] World Health Organization WHO Director-General’s Opening Remarks at the Media Briefing on COVID-19–11 March 2020. https://www.who.int/director-general/speeches/detail/who-director-general-s-opening-remarks-at-the-media-briefing-on-covid-19---11-march-2020.

[3] Shi S., Qin M., Shen B., Cai Y., Liu T., Yang F., Gong W., Liu X., Liang J., Zhao Q. (2020). Association of Cardiac Injury with Mortality in Hospitalized Patients With COVID-19 in Wuhan, China. JAMA Cardiol..

[4] Shi Y., Wang G., Cai X.P., Deng J.W., Zheng L., Zhu H.H., Zheng M., Yang B., Chen Z. (2020). An overview of COVID-19. J. Zhejiang Univ. Sci. B.

[5] World Health Organization SARS (Severe Acute Respiratory Syndrome). https://www.who.int/ith/diseases/sars/en.

[6] World Health Organization Middle East Respiratory Syndrome (MERS). https://www.cdc.gov/coronavirus/mers/index.html.

[7] Chan J.F., Yuan S., Kok K.H., To K.K., Chu H., Yang J., Xing F., Liu J., Yip C.C., Poon R.W. (2020). A familial cluster of pneumonia associated with the 2019 novel coronavirus indicating person-to-person transmission: A study of a family cluster. Lancet.

[8] Zhou P., Yang X.L., Wang X.G., Hu B., Zhang L., Zhang W., Si H.-R., Zhu Y., Li B., Huang C.-L. (2020). A pneumonia outbreak associated with a new coronavirus of probable bat origin. Nature.

[9] Huang Y., Yang C., Xu X.F., Xu W., Liu S.W. (2020). Structural and functional properties of SARS-CoV-2 spike protein: Potential antivirus drug development for COVID-19. Acta Pharmacol. Sin..

[10] Esakandari H., Nabi-Afjadi M., Fakkari-Afjadi J., Farahmandian N., Miresmaeili S.M., Bahreini E. (2020). A comprehensive review of COVID-19 characteristics. Biol. Proced. Online.

[11] Rahman H.S., Aziz M.S., Hussein R.H., Othman H.H., Omer S.H.S., Khalid E.S., Abdulrahman N.A., Amin K., Abdullah R. (2020). The transmission modes and sources of COVID-19, A systematic review. Int. J. Surg. Open.

[12] Yuen K.S., Ye Z.W., Fung S.Y., Chan C.P., Jin D.Y. (2020). SARS-CoV-2 and COVID-19, The most important research questions. Cell Biosci..

[13] Lovato A., de Filippis C., Marioni G. (2020). Upper airway symptoms in coronavirus disease 2019 (COVID-19). Am. J. Otolaryngol..

[14] Oran D.P., Topol E.J. (2020). Prevalence of Asymptomatic SARS-CoV-2 Infection: A Narrative Review. Ann. Internet Med..

[15] Paranjpe I., Russak A., De Freitas J.K., Lala A., Miotto R., Vaid A., Johnson K.W., Danieletto M., Golden E., Meyer D. (2020). Clinical Characteristics of Hospitalized Covid-19 Patients in New York City. medRxiv.

[16] Zhu J., Zhong Z., Ji P., Li H., Li B., Pang J., Zhang J., Zhao C. (2020). Clinicopathological characteristics of 8697 patients with COVID-19 in China: A meta-analysis. Fam. Med. Commun. Health.

[17] Song F., Shi N., Shan F., Zhang Z., Shen J., Lu H., Ling Y., Jiang Y., Shi Y. (2020). Emerging 2019 Novel Coronavirus (2019-nCoV) Pneumonia. Radiology.

[18] Xu Z., Shi L., Wang Y., Zhang J., Huang L., Zhang C., Liu S., Zhao P., Liu H., Zhu L. (2020). Pathological findings of COVID-19 associated with acute respiratory distress syndrome. Lancet Respir. Med..

[19] Tian S., Xiong Y., Liu H., Niu L., Guo J., Liao M., Xiao S.-Y. (2020). Pathological study of the 2019 novel coronavirus disease (COVID-19) through postmortem core biopsies. Mod. Pathol..

[20] Xiong T.Y., Redwood S., Prendergast B., Chen M. (2020). Coronaviruses and the cardiovascular system: Acute and long-term implications. Eur. Heart J..

[21] Kang Y., Chen T., Mui D., Ferrari V., Jagasia D., Scherrer-Crosbie M., Chen Y., Han Y. (2020). Cardiovascular manifestations and treatment considerations in COVID-19. Heart.

[22] Richardson P., McKenna W., Bristow M., Maisch B., Mautner B., O’Connell J., Olsen E., Thiene G., Goodwin J., Gyarfas I. (1996). Report of the 1995 World Health Organization/International Society and Federation of Cardiology Task Force on the Definition and Classification of cardiomyopathies. Circulation.

[23] Caforio A.L., Pankuweit S., Arbustini E., Basso C., Gimeno-Blanes J., Felix S.B., Fu M., Heliö T., Heymans S., Jahns R. (2013). Current state of knowledge on aetiology, diagnosis, management, and therapy of myocarditis: A position statement of the European Society of Cardiology Working Group on Myocardial and Pericardial Diseases. Eur. Heart J..

[24] Kociol R.D., Cooper L.T., Fang J.C., Moslehi J.J., Pang P.S., Sabe M.A., Shah R.V., Sims D.B., Thiene G., Vardeny O. (2020). Recognition and Initial Management of Fulminant Myocarditis: A Scientific Statement from the American Heart Association. Circulation.

[25] Ukimura A., Izumi T., Matsumori A. (2010). Clinical Research Committee on Myocarditis Associated with Influenza APiJobJCS. A national survey on myocarditis associated with the 2009 influenza A (H1N1) pandemic in Japan. Circ. J..

[26] Alhogbani T. (2016). Acute myocarditis associated with novel Middle east respiratory syndrome coronavirus. Ann. Saudi Med..

[27] Esfandiarei M., McManus B.M. (2008). Molecular biology and pathogenesis of viral myocarditis. Ann. Rev. Pathol..

[28] Woodruff J.F., Woodruff J.J. (1974). Involvement of T lymphocytes in the pathogenesis of coxsackie virus B3 heart disease. J. Immunol..

[29] Opavsky M.A., Penninger J., Aitken K., Wen W.H., Dawood F., Mak T., Liu P. (1999). Susceptibility to myocarditis is dependent on the response of alphabeta T lymphocytes to coxsackieviral infection. Circ. Res..

[30] Parrillo J.E., Cunnion R.E., Epstein S.E., Parker M.M., Suffredini A.F., Brenner M., Schaer G.L., Palmeri S.T., Cannon R.O., Alling D. (1989). A prospective, randomized, controlled trial of prednisone for dilated cardiomyopathy. N. Engl. J. Med..

[31] Mason J.W., O’Connell J.B., Herskowitz A., Rose N.R., McManus B.M., Billingham M.E., Moon T.E., Myocarditis Treatment Trial Investigators (1995). A clinical trial of immunosuppressive therapy for myocarditis. The Myocarditis Treatment Trial Investigators. N. Engl. J. Med..

[32] Hia C.P., Yip W.C., Tai B.C., Quek S.C. (2004). Immunosuppressive therapy in acute myocarditis: An 18 year systematic review. Arch. Dis. Child..

[33] Nishio R., Matsumori A., Shioi T., Ishida H., Sasayama S. (1999). Treatment of experimental viral myocarditis with interleukin-10. Circulation.

[34] Kuhl U., Pauschinger M., Schwimmbeck P.L., Seeberg B., Lober C., Noutsias M., Poller W., Schultheiss H.-P. (2003). Interferon-beta treatment eliminates cardiotropic viruses and improves left ventricular function in patients with myocardial persistence of viral genomes and left ventricular dysfunction. Circulation.

[35] Huber S.A. (1997). Autoimmunity in myocarditis: Relevance of animal models. Clin. Immunol. Immunopathol..

[36] Gauntt C.J., Arizpe H.M., Higdon A.L., Wood H.J., Bowers D.F., Rozek M.M., Crawley R. (1995). Molecular mimicry, anti-coxsackievirus B3 neutralizing monoclonal antibodies, and myocarditis. J. Immunol..

[37] Blauwet L.A., Cooper L.T. (2010). Myocarditis. Prog. Cardiovasc. Dis..

[38] Riski H., Hovi T., Frick M.H. (1980). Carditis associated with coronavirus infection. Lancet.

[39] Nakagawa K., Narayanan K., Wada M., Makino S. (2018). Inhibition of Stress Granule Formation by Middle East Respiratory Syndrome Coronavirus 4a Accessory Protein Facilitates Viral Translation, Leading to Efficient Virus Replication. J. Virol..

[40] Narayanan K., Huang C., Lokugamage K., Kamitani W., Ikegami T., Tseng C.T., Makino S. (2008). Severe acute respiratory syndrome coronavirus nsp1 suppresses host gene expression, including that of type I interferon, in infected cells. J. Virol..

[41] Lindner D., Fitzek A., Brauninger H., Aleshcheva G., Edler C., Meissner K., Scherschel K., Kirchhof P., Escher F., Schultheiss H.-P. (2020). Association of Cardiac Infection With SARS-CoV-2 in Confirmed COVID-19 Autopsy Cases. J. AMA Cardiol..

[42] Pirzada A., Mokhtar A.T., Moeller A.D. (2020). COVID-19 and Myocarditis: What Do We Know So Far?. CJC Open.

[43] Fairweather D., Cooper L.T., Blauwet L.A. (2013). Sex and gender differences in myocarditis and dilated cardiomyopathy. Curr. Probl. Cardiol..

[44] Zhou F., Yu T., Du R., Fan G., Liu Y., Liu Z., Xiang J., Wang Y., Song B., Gu X. (2020). Clinical course and risk factors for mortality of adult inpatients with COVID-19 in Wuhan, China: A retrospective cohort study. Lancet.

[45] Ukena C., Mahfoud F., Kindermann I., Kandolf R., Kindermann M., Bohm M. (2011). Prognostic electrocardiographic parameters in patients with suspected myocarditis. Eur. J. Heart Fail..

[46] Angeli F., Spanevello A., De Ponti R., Visca D., Marazzato J., Palmiotto G., Feci D., Reboldi G., Fabbri L.M., Verdecchia P. (2020). Electrocardiographic features of patients with COVID-19 pneumonia. Eur. J. Internet Med..

[47] He J., Wu B., Chen Y., Tang J., Liu Q., Zhou S., Chen C., Qin Q., Huang K., Lv J. (2020). Characteristic Electrocardiographic Manifestations in Patients With COVID-19. Can. J. Cardiol..

[48] Ferreira V.M., Schulz-Menger J., Holmvang G., Kramer C.M., Carbone I., Sechtem U., Kindermann I., Gutberlet M., Cooper L.T., Liu P. (2018). Cardiovascular Magnetic Resonance in Nonischemic Myocardial Inflammation: Expert Recommendations. J. Am. Coll. Cardiol..

[49] Luetkens J.A., Isaak A., Zimmer S., Nattermann J., Sprinkart A.M., Boesecke C., Rieke G.J., Zachoval C., Heine A., Velten M. (2020). Diffuse Myocardial Inflammation in COVID-19 Associated Myocarditis Detected by Multiparametric Cardiac Magnetic Resonance Imaging. Circ. Cardiovasc. Imaging.

[50] Aretz H.T., Billingham M.E., Edwards W.D., Factor S.M., Fallon J.T., Fenoglio J.J., Olsen E.G., Schoen F.J. (1987). Myocarditis. A histopathologic definition and classification. Am. J. Cardiovasc. Pathol..

[51] Chow L.H., Radio S.J., Sears T.D., McManus B.M. (1989). Insensitivity of right ventricular endomyocardial biopsy in the diagnosis of myocarditis. J. Am. Coll. Cardiol..

[52] Hauck A.J., Kearney D.L., Edwards W.D. (1989). Evaluation of postmortem endomyocardial biopsy specimens from 38 patients with lymphocytic myocarditis: Implications for role of sampling error. Mayo Clin. Proc..

[53] Dennert R., Crijns H.J., Heymans S. (2008). Acute viral myocarditis. Eur. Heart J..

[54] Cardiology E.S.O. ESC Guidance for the Diagnosis and Management of CV Disease during the COVID-19 Pandemic. https://www.escardio.org/Education/COVID-19-and-Cardiology/ESC-COVID-19-Guidance.

[55] Escher F., Pietsch H., Aleshcheva G., Bock T., Baumeier C., Elsaesser A., Wenzel P., Hamm C., Westenfeld R., Schiltheiss M. (2020). Detection of viral SARS-CoV-2 genomes and histopathological changes in endomyocardial biopsies. ESC Heart Fail..

[56] Dolhnikoff M., Ferranti J.F., de Almeida M.R.A., Duarte-Neto A.N., Gomes-Gouvea M.S., Degaspare N.V., Delgado A.F., Fiorita C.M., Leal G.N., Rodrigues R.M. (2020). SARS-CoV-2 in cardiac tissue of a child with COVID-19-related multisystem inflammatory syndrome. Lancet Child. Adolesc. Health.

[57] Halushka M.K., Vander Heide R.S. (2020). Myocarditis is rare in COVID-19 autopsies: Cardiovascular findings acrros 277 postmortem examinations. Cardiovasc. Pathol..

[58] Beigel J.H., Tomashek K.M., Dodd L.E., Mehta A.K., Zingman B.S., Kalil A.C., Hohmann E., Chu H.Y., Luetkemeyer A., Kiline S. (2020). Remdesivir for the Treatment of Covid-19-Final Report. N. Engl. J. Med..

[59] Hung I.F., Lung K.C., Tso E.Y., Liu R., Chung T.W., Chu M.Y., Ng Y.Y., Lo J., Chan J., Chung T.W. (2020). Triple combination of interferon beta-1b, lopinavir-ritonavir, and ribavirin in the treatment of patients admitted to hospital with COVID-19, an open-label, randomised, phase 2 trial. Lancet.

[60] Rajendran K., Krishnasamy N., Rangarajan J., Rathinam J., Natarajan M., Ramachandran A. (2020). Convalescent plasma transfusion for the treatment of COVID-19, Systematic review. J. Med. Virol..

[61] Johnson R.M., Vinetz J.M. (2020). Dexamethasone in the management of covid-19. BMJ.

[62] Group R.C., Horby P., Mafham M., Linsell L., Bell J.L., Staplin N., Emberson J.R., Wiselka M., Ustianowski A., Elmahi E. (2020). Effect of Hydroxychloroquine in Hospitalized Patients with Covid-19. N. Engl. J. Med..

[63] Kuster G.M., Pfister O., Burkard T., Zhou Q., Twerenbold R., Haaf P., Widmer A.F., Osswald S. (2020). SARS-CoV2, should inhibitors of the renin-angiotensin system be withdrawn in patients with COVID-19?. Eur. Heart J..

[64] Mehra M.R., Desai S.S., Kuy S., Henry T.D., Patel A.N. (2020). Cardiovascular Disease, Drug Therapy, and Mortality in Covid-19. N. Engl. J. Med..

[65] Reynolds H.R., Adhikari S., Pulgarin C., Troxel A.B., Iturrate E., Johnson S.B., Hausvater A., Newman J.D., Berger J.S., Bangalore S. (2020). Renin-Angiotensin-Aldosterone System Inhibitors and Risk of Covid-19. N. Engl. J. Med..

[66] Vaduganathan M., Vardeny O., Michel T., McMurray J.J.V., Pfeffer M.A., Solomon S.D. (2020). Renin-Angiotensin-Aldosterone System Inhibitors in Patients with Covid-19. N. Engl. J. Med..

[67] Inciardi R.M., Lupi L., Zaccone G., Italia L., Raffo M., Tomasoni D., Cani D.S., Cerini M., Farina D., Gavazzi E. (2020). Cardiac Involvement in a Patient with Coronavirus Disease 2019 (COVID-19). JAMA Cardiol..

[68] Mercuro N.J., Yen C.F., Shim D.J., Maher T.R., McCoy C.M., Zimetbaum P.J., Gold H.S. (2020). Risk of QT Interval Prolongation Associated with Use of Hydroxychloroquine with or Without Concomitant Azithromycin Among Hospitalized Patients Testing Positive for Coronavirus Disease 2019 (COVID-19). JAMA Cardiol..

[69] Holm A.M., Mehra M.R., Courtwright A., Teuteberg J., Sweet S., Potena L., Singer L.G., Farrero M., Shullo M.A., Benza R. (2020). Ethical considerations regarding heart and lung transplantation and mechanical circulatory support during the COVID-19 pandemic: An ISHLT COVID-19 Task Force statement. J. Heart Lung. Transpl..

[70] Chow J., Alhussaini A., Calvillo-Arguelles O., Billia F., Luk A. (2020). Cardiovascular Collapse in COVID-19 Infection: The Role of Venoarterial Extracorporeal Membrane Oxygenation (VA-ECMO). CJC Open.

[71] Document E.G. ELSO Guidance Document: ECMO for COVID-19 Patients with Severe Cardiopulmonary Failure. https://www.elso.org/Portals/0/Files/pdf/ECMO%20for%20COVID%2019%20Guidance%20Document.Final%2003.24.2020.pdf.

[72] Guo T., Fan Y., Chen M., Wu X., Zhang L., He T., Wang H., Wan J., Wang X., Lu Z. (2020). Cardiovascular Implications of Fatal Outcomes of Patients with Coronavirus Disease 2019 (COVID-19). JAMA Cardiol..

